# Toward Precision Acupuncture for Pain: Host Genetic Variability, Omics Biomarkers, and Treatment‐Response Stratification

**DOI:** 10.1155/humu/1645398

**Published:** 2026-05-31

**Authors:** Lianying Chang, Yue Pan, Xu He, Jin Yang, Lei Xiao, Jieying Zhang, Shuang He, Yanlong Xie

**Affiliations:** ^1^ First Teaching Hospital of Tianjin University of Traditional Chinese Medicine, Tianjin, China, tjtcm.cn; ^2^ National Clinical Research Center for Chinese Medicine, Tianjin, China; ^3^ Sichuan Orthopedic Hospital, Chengdu, Sichuan, China; ^4^ State Key Laboratory of Chinese Medicine Modernization, Tianjin University of Traditional Chinese Medicine, Tianjin, China, tjutcm.edu.cn; ^5^ Tianjin Academy of Traditional Chinese Medicine Affiliated Hospital, Tianjin, China; ^6^ Tianjin Academy of Traditional Chinese Medicine, Tianjin, China

**Keywords:** acupuncture, chronic pain, genetic polymorphism, multiomics, precision medicine, predictive biomarkers, treatment-response stratification

## Abstract

Pain is a heterogeneous clinical condition characterized by substantial interindividual variability in symptom severity and treatment response. Acupuncture has been widely used for the management of various pain disorders, including chronic musculoskeletal pain, migraine, and cancer‐related pain. However, clinical outcomes remain highly variable across patients, suggesting that average treatment effects may not fully capture biologically and clinically meaningful response heterogeneity. Recent advances in human genetics and multiomics technologies have provided new opportunities to investigate the biological factors that may contribute to this variability. Current genetic evidence, derived mainly from candidate‐gene studies, suggests that polymorphisms involved in pain perception and neuromodulatory pathways, including COMT and OPRM1, may influence individual sensitivity to acupuncture analgesia; however, these findings remain exploratory and require validation in larger and more diverse cohorts. In parallel, transcriptomic, epigenetic, proteomic, metabolomic, and inflammatory profiling studies have identified molecular changes associated with acupuncture treatment. These treatment‐associated signals should be distinguished from predictive biomarkers: Baseline genetic or molecular features may help estimate the likelihood of response, whereas posttreatment molecular alterations more often reflect treatment engagement, biological adaptation, or downstream mechanistic effects. Although the available evidence remains fragmented and is often limited by small sample sizes, heterogeneous acupuncture protocols, variable analytical pipelines, and insufficient external validation, it provides a useful foundation for developing biomarker‐informed approaches to acupuncture research. In this review, we summarize current evidence linking host genetic variability and omics‐derived molecular signatures to acupuncture analgesia, clarify the conceptual distinction between predictive and treatment‐associated biomarkers, and discuss the potential and limitations of response‐stratified acupuncture. We further highlight key priorities for the field, including standardized treatment protocols, multicenter cohorts, prospective biospecimen collection, reproducible omics workflows, and external validation of prediction models. Together, these considerations support precision acupuncture as an emerging research framework for understanding and eventually improving individualized pain management, rather than as a currently established clinical strategy.

## 1. Introduction

Pain is one of the leading causes of disability worldwide and remains a major clinical and public health challenge [1, 2]. Chronic pain conditions, including musculoskeletal pain, migraine, neuropathic pain, and cancer‐related pain, affect large populations and impose substantial socioeconomic and healthcare burdens [3, 4]. Although pharmacological therapies such as nonsteroidal anti‐inflammatory drugs, opioids, and adjuvant analgesics remain central to pain management, their long‐term use is frequently constrained by incomplete efficacy, adverse effects, interindividual variability in drug response, and concerns regarding dependence or tolerance [5–7]. These limitations have encouraged increasing interest in nonpharmacological strategies that may complement conventional pain management and reduce reliance on long‐term pharmacotherapy [8].

Acupuncture is one of the most widely used nonpharmacological interventions for pain control and has been evaluated across diverse pain‐related conditions [9]. Randomized trials and meta‐analyses have suggested that acupuncture may provide modest but meaningful benefit in disorders such as chronic low back pain, osteoarthritis, migraine, and tension‐type headache [10–13]. At the same time, experimental studies have indicated that acupuncture analgesia may involve multiple biological processes, including modulation of endogenous opioid activity, neurotransmitter signaling, and neuroimmune or inflammatory pathways [14–16]. Together, these observations support the view that acupuncture exerts biologically measurable effects rather than functioning solely as a nonspecific intervention [17].

A major unresolved issue is the marked heterogeneity of clinical response to acupuncture. In both clinical practice and research settings, some patients experience substantial and sustained pain relief, whereas others derive only limited benefit despite receiving broadly similar interventions [18, 19]. This variability is increasingly recognized as a central issue in acupuncture research, because analyses based only on average treatment effects may obscure biologically meaningful differences between patient subgroups [18, 20]. Understanding the determinants of this response heterogeneity is therefore essential for improving the clinical utility of acupuncture and for moving beyond empiric treatment selection [21].

Recent advances in human genetics and high‐throughput molecular profiling provide new opportunities to investigate why patients differ in pain sensitivity and treatment responsiveness [22, 23]. In broader pain research, genetic variation in catecholaminergic signaling, opioid pathways, ion‐channel function, and inflammatory regulation has been linked to interindividual differences in nociception and analgesic response [24, 25]. However, translating these observations into acupuncture research requires caution. Evidence directly linking host genetic variation to acupuncture responsiveness remains preliminary, and most available studies are based on candidate genes rather than large‐scale genomic discovery or independent validation cohorts. Thus, genetic markers should currently be viewed as biologically plausible candidates rather than established predictors of acupuncture outcome.

Multiomics approaches offer another route for characterizing the biological effects of acupuncture. Transcriptomic, epigenetic, proteomic, metabolomic, and inflammatory profiling studies have reported treatment‐associated molecular alterations in experimental models and selected human pain cohorts [17, 26, 27]. A key conceptual distinction is necessary at this point. Baseline or pretreatment features, such as host genetic variants or molecular profiles, may function as candidate predictive biomarkers if they are associated with subsequent clinical benefit. By contrast, molecular changes observed during or after acupuncture more often represent treatment‐associated biological responses, treatment engagement, or downstream physiological adaptation. Conflating these two categories may lead to overinterpretation of mechanistic or pharmacodynamic‐like signals as clinically actionable predictors.

**Figure 1:**
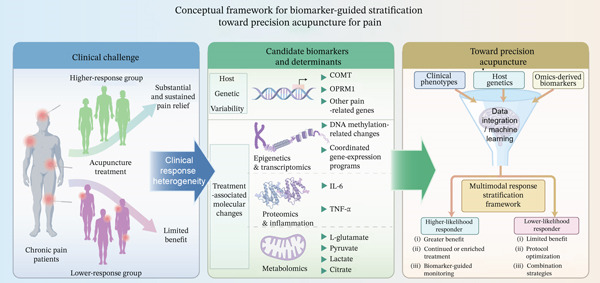
Conceptual framework for biomarker‐guided stratification toward precision acupuncture for pain. Heterogeneous clinical responses to acupuncture may be associated with multiple candidate determinants, including host genetic variability, baseline clinical phenotypes, and treatment‐associated multiomics changes. Integration of clinical phenotypes, host genetics, and omics‐derived molecular signatures may support multimodal response stratification and inform more individualized acupuncture research strategies.

Within this context, precision medicine provides a useful framework for rethinking acupuncture‐based pain management, but its application remains at an early stage [21, 28]. Rather than assuming uniform benefit across patients, a precision‐oriented approach seeks to integrate clinical phenotypes, host biological factors, and molecular readouts to identify patient subgroups with different probabilities of response [21, 22]. For acupuncture, this framework should be understood as an emerging research paradigm rather than a mature clinical strategy. Its development will require standardized acupuncture protocols, harmonized outcome definitions, adequately powered multicenter cohorts, reproducible omics workflows, and prospective biomarker validation. In this review, we summarize current evidence linking host genetic variability and omics‐derived molecular signatures to acupuncture analgesia, clarify the distinction between predictive biomarkers and treatment‐associated molecular changes, and discuss how these emerging data may inform future biomarker‐guided treatment‐response stratification in pain management (Figure 1).

## 2. Genetic Variability in Pain Susceptibility and Analgesic Response

Interindividual variability in pain perception and analgesic response is a well‐recognized feature of pain biology [29, 30]. Even under comparable nociceptive stimulation or treatment conditions, individuals may differ substantially in pain sensitivity, chronic pain susceptibility, and therapeutic outcomes [31]. Genetic variation is one contributor to this variability, although its effects are usually modest, context‐dependent, and influenced by environmental and clinical factors. Over the past two decades, accumulating evidence has shown that host genetic variation contributes to these differences and helps shape the biological heterogeneity observed across pain phenotypes [32].

One of the most frequently investigated genes in this field is catechol‐O‐methyltransferase (COMT), which encodes an enzyme involved in the degradation of catecholamines, including dopamine, epinephrine, and norepinephrine [33]. Through its influence on catecholaminergic tone, COMT may affect descending pain modulation, stress responsiveness, and central nociceptive processing [34]. The Val158Met polymorphism, rs4680, has been associated with experimental pain sensitivity, chronic pain risk, and variability in analgesic response in several studies [31, 33, 34]. However, COMT should be interpreted as a representative pain‐modulatory candidate gene rather than a universal determinant of pain or treatment response. Reported associations vary across pain phenotypes, study designs, ethnic backgrounds, and clinical contexts, indicating that its predictive value remains limited when considered in isolation.

OPRM1, which encodes the *μ*‐opioid receptor, represents another biologically plausible gene because of its central role in endogenous and exogenous opioid‐mediated analgesia [35]. The A118G polymorphism, rs1799971, has been linked to altered receptor function, opioid requirements, placebo responsiveness, and pain sensitivity [31, 36]. These observations are relevant to acupuncture research because endogenous opioid pathways are among the proposed mechanisms of acupuncture analgesia. Nevertheless, most evidence regarding OPRM1 comes from broader pain, opioid pharmacogenetic, or placebo‐response studies rather than from acupuncture‐specific cohorts. Therefore, its relevance to acupuncture responsiveness remains biologically plausible but not yet clinically established.

Beyond COMT and OPRM1, additional genes involved in nociceptive transmission and pain amplification have been investigated. TRPV1 participates in thermal and inflammatory nociception, SCN9A encodes the Nav1.7 sodium channel that is critical for peripheral pain signaling, and cytokine‐related loci may influence inflammatory pain states [37–39]. These genes support the broader principle that pain susceptibility is shaped by multiple biological systems rather than by a single pathway. However, they should be clearly distinguished from validated acupuncture‐response biomarkers. At present, their role in acupuncture research is mainly indirect, offering mechanistic plausibility and candidate pathways for future investigation rather than direct evidence of treatment‐response prediction.

Taken together, current pain‐genetics evidence suggests that inherited biological differences can contribute to variability in pain susceptibility and analgesic response [37]. This background provides a rationale for examining whether host genetic factors may also influence response heterogeneity in nonpharmacological interventions such as acupuncture. However, extrapolation from general pain genetics to acupuncture responsiveness must be made cautiously. Clinically useful genetic predictors of acupuncture analgesia would require dedicated acupuncture‐treated cohorts, standardized outcome definitions, adequate sample sizes, and independent validation [40, 41]. Accordingly, the existing pain‐genetics literature should be viewed as a conceptual foundation for precision acupuncture research, rather than as definitive evidence that specific genetic variants can currently guide acupuncture treatment selection.

## 3. Genetic Evidence Directly Related to Acupuncture Response

Compared with the broader literature on pain genetics, evidence directly linking host genetic variation to acupuncture responsiveness remains scarce and preliminary [25, 41]. Existing studies have mainly adopted candidate‐gene approaches, usually in relatively small cohorts and within specific clinical contexts, such as chronic pain, cancer survivorship, or electroacupuncture‐based interventions [25, 28]. This design has provided useful early signals, but it also limits the strength, reproducibility, and generalizability of the conclusions. Therefore, the current literature should be interpreted as an initial attempt to test whether acupuncture response has a heritable or biologically stratifiable component, rather than as evidence that genetic markers can already guide clinical treatment selection.

Among the candidate loci evaluated, COMT has received the greatest attention. Because of its established role in catecholamine metabolism and central pain regulation, the Val158Met (rs4680) variant has been investigated as a potential modifier of acupuncture‐induced analgesia. Several studies have suggested that this polymorphism may be associated with differential treatment benefit in specific pain settings, although the reported direction and magnitude of the association have not been fully consistent across cohorts [25, 28]. These findings should therefore be interpreted cautiously, but they nonetheless support the plausibility that variation in catecholaminergic signaling may influence acupuncture responsiveness.

Other genes, particularly those related to endogenous opioid signaling, have also been proposed as potential contributors. Experimental studies have shown that acupuncture can engage opioid‐mediated analgesic mechanisms, making genes such as OPRM1 biologically relevant candidates [42]. However, compared with COMT, direct clinical evidence for these loci in acupuncture‐treated populations remains sparse. At present, the main value of this literature lies less in establishing definitive predictive markers than in demonstrating that acupuncture response is a biologically testable trait rather than a purely empirical clinical observation.

Overall, current genetic evidence provides a plausible but not definitive basis for considering host genotype as one contributor to acupuncture‐response heterogeneity. The main value of this literature is not that it has already identified clinically actionable predictors, but that it supports acupuncture responsiveness as a biologically testable phenotype. Future studies should move beyond isolated candidate‐gene analyses and incorporate larger, well‐phenotyped, multicenter cohorts with standardized acupuncture protocols, harmonized outcome measures, and independent validation. Only through such designs will it be possible to determine whether host genetic variation can meaningfully contribute to biomarker‐guided stratification in acupuncture‐based pain management.

## 4. Omics Evidence Associated With Acupuncture Analgesia

### 4.1. Transcriptomic and Epigenetic Signals Associated With Acupuncture Analgesia

Compared with metabolomic and targeted biomarker studies, transcriptomic evidence in acupuncture analgesia remains relatively limited and is derived more often from experimental models than from large human pain cohorts [26, 43]. Nevertheless, existing studies suggest that acupuncture can influence coordinated gene expression programs related to neuroimmune regulation, inflammatory signaling, and synaptic plasticity, rather than acting through a single downstream pathway [44, 45]. Emerging work has also extended this framework to epigenetic regulation, including DNA methylation‐related changes observed in neuropathic pain models, supporting the possibility that acupuncture‐induced analgesia involves broader transcriptional and regulatory remodeling [46–48]. At present, however, these findings are best regarded as mechanistic clues rather than clinically actionable biomarkers, given the limited cross‐cohort validation in human pain populations.

### 4.2. Proteomic and Inflammatory Biomarker Evidence

Proteomic and targeted biomarker studies provide more direct evidence that acupuncture is accompanied by measurable molecular changes relevant to pain biology [49]. In migraine, plasma proteomic analyses performed before and after acupuncture have identified differential proteins and pathway‐level changes related to immune responses, arginine biosynthesis, glycolysis/gluconeogenesis, and riboflavin metabolism [50]. Integrative work combining proteomics, metabolomics, and functional neuroimaging has further suggested that acupuncture and sham acupuncture may engage distinct biological patterns, with acupuncture more closely linked to networks involving hypoxic‐stress responses, inflammation regulation, and restoration of brain energy imbalance [17, 50]. In parallel, clinical studies have frequently evaluated inflammatory mediators such as IL‐6 and TNF‐*α* as treatment‐associated signals [51, 52]. Although these markers cannot yet be regarded as validated predictors of response, they support the view that acupuncture analgesia is accompanied by measurable neuroimmune and inflammatory changes.

### 4.3. Metabolomic Signatures Associated With Acupuncture Treatment

Among currently available omics approaches, metabolomics offers some of the strongest acupuncture‐specific human evidence in pain research [50]. A recent systematic review identified a growing number of human studies examining metabolomic changes after acupuncture, while also emphasizing that the field remains at an early stage overall [26, 50]. Within pain‐related conditions, migraine has emerged as one of the most informative clinical settings [17, 50]. Plasma metabolomic studies have reported acupuncture‐associated alterations in pathways related to energy metabolism, amino acid turnover, and lipid regulation, including changes involving metabolites such as L‐glutamate, pyruvate, lactate, and citrate [50, 53]. These findings are of particular interest because they point to potential modulation of anaerobic glycolysis and mitochondrial energy metabolism, providing a biochemical framework through which acupuncture‐related symptom improvement might be interpreted [50, 54]. Although the reported signatures vary across studies and disease contexts, metabolomic profiling appears especially promising for capturing treatment‐related biological shifts and nominating candidate biomarkers for future response stratification.

## 5. Toward Biomarker‐Guided Precision Acupuncture

### 5.1. Genetic Information for Response Stratification

A logical first step toward precision acupuncture is the incorporation of host genetic information into treatment stratification strategies [41]. Early exploratory studies suggest that inherited variation in neuromodulatory pathways may contribute to differential clinical responses to acupuncture‐based pain interventions [25]. Among the candidate loci examined, COMT Val158Met has received the greatest attention, with several studies reporting associations between this variant and differences in pain improvement after acupuncture in chronic pain settings, including musculoskeletal pain in cancer survivors [25, 28]. Although these findings remain preliminary and require replication in larger and more diverse cohorts, they illustrate how host genotype could potentially be used to identify subgroups with differing probabilities of therapeutic benefit. At this stage, however, COMT and other candidate loci should be regarded as exploratory markers rather than validated predictors of acupuncture efficacy. In the longer term, broader genomic approaches may help move the field beyond isolated candidate genes toward a more systematic understanding of genetic contributions to acupuncture responsiveness. Such progress will require adequately powered cohorts, standardized response definitions, and independent validation across different pain conditions and populations.

### 5.2. Candidate Biomarkers for Monitoring Treatment Engagement

In addition to inherited variation, biological measurements obtained during or after treatment may provide useful information about how patients engage with acupuncture at the molecular level [26, 50]. Importantly, these markers should not yet be viewed as established predictors of treatment success; rather, they are better considered candidate response markers or indicators of treatment‐related biological activity [53]. This distinction is important because predictive biomarkers should ideally be measured before treatment and associated with subsequent clinical benefit, whereas many omics signals reported after acupuncture reflect treatment‐associated molecular changes. Studies in chronic pain and migraine have shown that acupuncture can be accompanied by changes in circulating inflammatory mediators, neuropeptides, and metabolic intermediates, some of which correlate with symptom improvement [16, 17, 50]. Metabolomic shifts involving energy metabolism, lipid regulation, and amino acid turnover, for example, may reflect systemic physiological adaptation to treatment rather than merely nonspecific background fluctuation. Such markers may therefore prove useful not only for mechanistic interpretation but also for monitoring treatment engagement in future biomarker‐guided frameworks. However, their role in patient selection will require prospective testing, reproducible measurement, and validation in independent cohorts.

### 5.3. Integrative Prediction Models for Acupuncture Responsiveness

The ultimate goal of precision acupuncture is to integrate clinical characteristics with genetic and molecular information in order to identify patients most likely to benefit from treatment [55]. Recent developments in computational biology and machine learning have made it increasingly feasible to analyze multidimensional datasets that combine symptom profiles, molecular biomarkers, neuroimaging signals, and other clinical variables [56]. Exploratory studies have already begun to apply such approaches to classify responders and nonresponders in chronic pain cohorts receiving acupuncture [55, 57, 58]. Although these efforts remain early‐stage and are often based on small datasets, they provide proof of concept that acupuncture responsiveness can be approached as a stratifiable biological trait rather than an exclusively empirical phenomenon [57]. Nevertheless, most current models should be interpreted as proof‐of‐concept rather than clinically deployable prediction tools because they are commonly limited by small sample sizes, heterogeneous endpoints, and insufficient external validation. With larger cohorts, standardized outcome definitions, and external validation, integrative models combining clinical phenotypes, host genetics, omics‐derived biomarkers, and imaging features may eventually support more reliable and clinically useful treatment‐response stratification. Before such models can inform clinical decision‐making, future studies will need transparent model reporting, prospective validation, and evaluation of whether prediction models improve upon simpler clinical stratification approaches.

## 6. Discussion

Collectively, the currently available evidence suggests that acupuncture analgesia should not be viewed as a biologically uniform phenomenon [43, 59]. Rather, the literature supports a model in which treatment response is shaped by multiple interacting factors, including baseline pain biology, host genetic variability, and downstream molecular changes associated with treatment exposure [25, 26, 60]. Although this evidence remains fragmented, it is sufficient to challenge the traditional assumption that acupuncture effects can be adequately understood through average treatment effects alone [55, 61]. From this perspective, the emerging concepts of response heterogeneity, biomarker‐guided stratification, and precision intervention provide a useful framework for reinterpreting acupuncture‐based pain management in a more biologically grounded manner [62].

At the same time, the current evidence base is still far from sufficient to support routine clinical implementation of precision acupuncture strategies [56, 62]. One major limitation is the small size and heterogeneity of most existing studies. Genetic investigations of acupuncture response have largely relied on candidate‐gene designs and relatively limited patient cohorts, whereas omics studies often involve specific disease settings, modest sample sizes, and variable analytical pipelines [26, 41]. This makes it difficult to determine whether reported associations are robust, generalizable, or disease‐specific. In addition, inconsistencies in acupuncture protocols, including point selection, stimulation method, treatment frequency, and practitioner‐dependent variation, further complicate comparisons across studies [63]. Such methodological heterogeneity may influence both clinical outcomes and molecular readouts, thereby limiting the reproducibility of biomarker discovery and the development of reliable response‐prediction models.

Another important issue is that not all biomarker signals identified in current studies should be interpreted as predictive markers [50]. Many of the molecular changes reported after acupuncture treatment, including inflammatory mediators, proteomic shifts, and metabolomic alterations, may reflect treatment engagement or downstream physiological adaptation rather than baseline determinants of response [53]. Distinguishing predictive biomarkers from pharmacodynamic or treatment‐associated readouts will therefore be essential in future work [26]. Similarly, although genes such as COMT provide a plausible starting point for understanding inherited variability in acupuncture response, the field has not yet established stable or independently validated genomic predictors [25, 41]. As a result, the translational significance of current findings should be interpreted with caution.

Reproducibility is another major challenge for omics‐based acupuncture research. Current studies differ substantially in study population, pain phenotype, disease duration, medication background, biospecimen type, sampling time point, omics platform, normalization method, batch‐effect correction, statistical threshold, and analytical workflow. These differences may partly explain why transcriptomic, proteomic, inflammatory, and metabolomic signatures are not yet consistent across studies. Greater transparency in preprocessing, feature selection, statistical modeling, and data sharing will be necessary to determine whether reported molecular signatures are reproducible and biologically meaningful. Without such methodological standardization, it will remain difficult to separate robust treatment‐associated biology from cohort‐specific or pipeline‐dependent findings.

Despite these limitations, the field is moving in a direction that is conceptually aligned with broader trends in precision medicine. Future studies will likely benefit from larger and better phenotyped cohorts, more standardized acupuncture protocols, and integrative designs that combine genomic data, omics‐derived biomarkers, neuroimaging measures, and detailed clinical phenotypes. Equally important will be the use of prospective validation strategies and external replication to determine whether candidate biomarkers can reliably classify responders and nonresponders across pain conditions. Priority should be given to multicenter cohorts, harmonized response definitions, predefined biomarker endpoints, standardized biospecimen collection, and transparent computational modeling. Machine learning models should also be externally validated and evaluated for interpretability, calibration, and clinical feasibility before being considered for decision support. If these challenges can be addressed, acupuncture research may gradually move from descriptive efficacy studies toward biologically informed treatment stratification, thereby improving both mechanistic understanding and clinical applicability.

## 7. Conclusion

Acupuncture analgesia is increasingly understood as a heterogeneous biological and clinical phenomenon rather than a uniform intervention effect. Current evidence suggests that host genetic variability and treatment‐associated molecular changes may help explain why patients differ in their response to acupuncture, but most available findings remain exploratory and insufficiently validated for clinical decision‐making. A key message of this review is that baseline predictive biomarkers should be distinguished from molecular changes observed during or after treatment, which more often reflect biological engagement or downstream adaptation. Future progress will depend on standardized acupuncture protocols, multicenter cohorts, reproducible omics workflows, and prospective validation of candidate biomarkers and prediction models. With these methodological advances, precision acupuncture may evolve from a conceptual framework into a more evidence‐based strategy for individualized pain management.

## Author Contributions

L.C. and Y.X. conceived and designed the study. L.C. wrote the manuscript. Y.P., X.H., J.Y., L.X., J.Z., and S.H. contributed to literature review, data interpretation, and manuscript revision. S.H. and Y.X. supervised the project.

## Funding

This study was supported by the Scientific Research Program Project of Hebei Administration of Traditional Chinese Medicine (T2025111).

## Disclosure

All authors read and approved the final manuscript.

## Conflicts of Interest

The authors declare no conflicts of interest.

## Data Availability

Data sharing is not applicable to this article as no datasets were generated or analyzed during the current study.
